# S1‐Guideline for diagnosis and therapy of necrobiosis lipoidica

**DOI:** 10.1111/ddg.15943

**Published:** 2025-12-19

**Authors:** Cornelia Erfurt‐Berge, Regina Renner, Melanie Peckruhn, Jörg Tittelbach, Dorothea Terhorst‐Molawi, Friederike Kauer, Joachim Dissemond

**Affiliations:** ^1^ Hautklinik Uniklinikum ErlangenFriedrich‐Alexander‐Universität Erlangen‐Nürnberg; ^2^ Practice Esslingen; ^3^ Department of Dermatology University Hospital Jena Friedrich Schiller University Jena; ^4^ Institute of Allergology Charité – Universitätsmedizin Berlin; ^5^ Immunology and Allergology Fraunhofer Institute for Translational Medicine and Pharmacology ITMP, Berlin; ^6^ Practice Potsdam; ^7^ Department of Dermatology Venereology and Allergology University Hospital Essen

**Keywords:** Diabetes mellitus, leg ulcer, necrobiosis lipoidica, quality of life, S1 guideline

## Abstract

Necrobiosis lipoidica (NL) is a rare granulomatous skin disease of unknown etiology that occurs frequently in association with diabetes mellitus and other comorbidities. The predilection site is the lower leg, particularly the pretibial areas. The exact pathogenesis remains unclear. Vascular disorders with microangiopathic changes and an autoimmune genesis are discussed. Necrobiosis lipoidica occurs three to six times more frequently in women. Men tend to show a more severe course and develop ulcerations more frequently. The diagnosis can often be established based on typical clinical and dermatoscopic findings. A biopsy should be performed in clinically unclear cases, in the presence of ulceration, or if there are signs of malignant transformation. Overall, the scientific data available for NL are still insufficient and there is a need for further research. However, as patients often experience a severely impaired quality of life, it is important to be aware of the limited scientific evidence and to translate it into practical therapeutic recommendations. This short version of the S1 guideline of the German Dermatological Society (DDG) summarizes the current evidence and, incorporating expert assessments, provides specific recommendations for everyday clinical practice.

## INTRODUCTION

Necrobiosis lipoidica (NL) is a rare granulomatous skin disease with unknown etiology (Orphanet Code: 542592). Reliable figures on its prevalence and incidence are not available. In patients with diabetes‐associated NL, the average age of manifestation is in the 3^rd^ and 4^th^ decade of life, while in patients without diabetes NL will be diagnosed in the 4^th^ or 5^th^ decade of life. Patient with Type‐1 diabetes will usually experience an earlier onset.^1^, ^2^ Necrobiosis lipoidica occurs three to six times more frequently in women than in men, but severe disease and ulceration is more common in men.^3^, ^4^, ^5^, ^6^

## PATHOGENESIS

The exact pathogenesis of NL remains unknown. Possible mechanisms that are currently discussed include vascular conditions with microangiopathic alterations and immune complex deposits, or pronounced collagen degeneration in the context of autoimmune disease.^7^, ^8^ Trauma as well as metabolic and inflammatory processes lead to activation and migration of neutrophils which dominate the inflammatory infiltrations in the early stage of the disease.^9^ Macrophage proliferation caused by the neutrophils leads to granuloma formation. It is unclear if there is a genetic predisposition, although there are a number of case reports on familial NL which may or may not be associated with diabetes mellitus.^9^, ^10^, ^11^, ^12^ The most common pathological vascular alterations are thickening of the vascular walls, fibrosis, and endothelial proliferation.^13^ Immunofluorescence diagnostics have shown deposits of fibrin, fibrinogen, immunoglobulins (mainly IgM), and complement factors (mainly C3) on the dermoepidermal junction of the blood vessels.^14^ Vessel thickening and the resulting increased activation of the coagulation system are caused by glycoprotein deposits detected in the vessel walls in patients with diabetes as well as patients with NL.^15^, ^16^ Increased occurrence of Glut‐1 receptors (human erythrocyte glucose transporter) in fibroblasts is being discussed as a possible factor influencing blood flow.^17^ The collagen concentration is decreased in NL. One topic of debate is increased lysyl oxidase levels, which are responsible for collagen crosslinking in diabetes patients. This leads to end organ damage, accelerated ageing, and also to the wax‐like thickening of vessel membranes typically found in NL.^18^

## DIAGNOSTICS AND CLINICAL COURSE

Necrobiosis lipoidica can frequently be diagnosed clinically due to its typical appearance. A careful anamnesis and if appropriate serological investigations can detect the first appearance of disease signs, its proliferation, comorbidities such as diabetes mellitus, and differentiation from similar conditions (differential diagnoses). The typical clinical appearance of NL shows oval plaques with brownish to brownish‐livid margins, while the center is initially reddish‐brown and later yellowish‐brown and atrophic. Telangiectasia is also typical.^19^ Dermatoscopy shows clearly delineated, elongated, and exceedingly tortuous telangiectasias on a whitish or yellowish‐orange structureless background.^20^, ^21^, ^22^ In the early stages, most patients experience hardly any symptoms, although pruritus, dysesthesia, or pain may occur.^23^ The lower legs are the typical predilection sites, especially the pretibial areas.^1^, ^2^ In about 7–15% of cases, the skin of the arms, abdomen, or genital area was affected, usually as additional lesions.^1^, ^24^, ^25^, ^26^ Skin lesions may occur individually, but multiple lesions are more common.^27^ The course of this disease is chronic. Ulceration may occur as a complication in up to 35% of patients, frequently after minor injuries.^3^, ^4^, ^28^ Risk factors for ulceration include male sex and (insufficiently controlled) diabetes mellitus.^29^ Another, rare complication is the development of cutaneous neoplasia, especially in cases of ulcerated NL, over a period of several years.^30^, ^31^, ^32^, ^33^ There are no specific laboratory parameters that can be used for NL diagnostics (Table 1).

**TABLE 1:** Recommendations for the diagnosis of necrobiosis lipoidica.

**Recommendation**	**Strength**
For the diagnosis of Necrobiosis lipoidica, *it is recommended* to consider the following aspects: *Medical History* – Occurs more frequently in women than in men – Typical first appearance in middle‐aged to elderly persons – Occurrence in children has been reported in patients with Type‐1 diabetes – Presence of an associated condition *Clinical Appearance* – Brownish‐yellow plaques, initially with inflamed margins and later with atrophic center – Frequently located on the lower legs, especially in the pretibial area – Ulceration may occur within the plaques Dermatoscopy *may be considered* as an additional diagnostic feature.	↑↑ 0
For the diagnosis of Necrobiosis lipoidica, a biopsy for histologic confirmation *is recommended* in clinically unclear cases, in the presence of ulceration, or if signs of malignant transformation are present.	↑↑

↑↑ – wird empfohlen, ↑ – kann empfohlen werden, 0 – kann erwogen werden

↑↑ – is recommended, ↑ – may be recommended, 0 – may be considered

## HISTOLOGY

Histological appearance varies, depending on duration, and is also influenced by the association of NL with diabetes mellitus.^34^ The epidermis appears mostly normal but may also be atrophic, acanthotic, covered by hyperkeratosis, or in some cases ulcerated. Underneath, the entire corium extending down to the subcutis and particularly into the connective tissue septa, shows palisade granulomas in diabetes patients while in non‐diabetic NL patients sarcoidal‐type granulomatous reactions tend to be seen.^35^ Early lesions display superficial or deep inflammatory infiltrations with mixed cell types or dominant neutrophils which may in some cases extend to the connective tissue septa or the subcutaneous fatty tissue. Necrotizing vasculitis near the necrobiosis zones as well as necrosis of adnexal structures may occur. Necrobiosis zones usually show horizontal orientation and linear alignment. They consist of eosinophilic, engorged, or degenerated collagen fibers which appear hyalinized and are surrounded by lymphocytes and histiocytes, usually with plasma cells in‐between. Polynuclear giant cells of the Langerhans or foreign body type may be abundant and are the dominant cell type in the connective tissue septa, particularly in the sarcoidal type of NL. In rare cases, the necrobiosis zones contain mucin and simultaneously display a loss of elastic fibers, which can be demonstrated with Elastica‐van‐Gieson staining.^36^ Vascular alterations such as vessel wall thickening, intimal proliferation, and lumen constriction with formation of thrombi may also occur. In long‐established lesions, the connective tissue is parallelized and fibrosed, and the collagen fibers are sclerotic with interspersed plasma cells. In late‐stage disease, elastic fibers are lost and can no longer be detected.^37^

If the clinical appearance is unambiguous, biopsy can be avoided since there is a high risk of impaired wound healing.^29^

## DIFFERENTIAL DIAGNOSES

Differential diagnoses include other diseases with similar clinical or histological features.^38^ (Table 2). In differentiation from annular granulomas, for example, dermatoscopy does not show telangiectasia but only structureless orange to reddish peripheral linear erythema.^20^ In rare cases, cutaneous sarcoidosis may show skin changes that appear similar to NL.^39^

**TABLE 2:** Differential diagnoses of necrobiosis lipoidica.

Major differential diagnoses for Necrobiosis lipoidica
Granuloma annulare
Cutaneous sarcoidosis
Necrobiotic xanthogranuloma
*If ulcerated*:
Venous leg ulcer
Arterial leg ulcer
Ulcerated neoplasia
Other causes for leg ulcers

Less common differential diagnoses
Morphea
Stasis dermatitis, purpura jaune d'ocre
Erythema nodosum
Erythema induratum
Lichen sclerosus et atrophicus
Radiodermatitis
Tertiary syphilis
Nodular panniculitis
Tuberculoid lepra

## ASSOCIATED DISEASES

One commonly associated disease, found in 11–65 % of NL patients, is diabetes mellitus.^1^, ^3^, ^4^, ^40^ NL may precede the diagnosis of diabetes, or be diagnosed simultaneously. In many NL patients, mostly those with type‐1 diabetes, blood glucose management is insufficient.^1^, ^41^ However, NL is found in less than 1% of all diabetes patients.^42^, ^43^ Other associated diseases include thyroid disorders in about 15–25% of NL patients, particularly hypothyreosis.^1^, ^29^ It is therefore recommended to conduct laboratory investigations for these conditions in NL patients. Improvement of NL lesions in type‐1 diabetes patients has been demonstrated after optimization of diabetes control.^44^ Consequently, there appears to be a causal link between a glycemic metabolic state and NL. The general effects of diabetes, in particular on wound healing, show optimization to be an advantageous factor, albeit not the only one. Other aspects of metabolic syndrome such as obesity, arterial hypertension, and lipid metabolism disorders also show increased association although a causal link has yet to be clearly established.^4^, ^45^

## TREATMENT

### Topical treatments

Topical treatments (Table 3) can be effective in the active inflammatory margins of the lesions. In many cases, the center of the skin lesion is already atrophic and shows a conversion to connective tissue with no detectable signs of inflammation. Topical anti‐inflammatory treatments in the center of the lesion are therefore usually not promising. High‐potency topical glucocorticoids as well as topical tacrolimus (off label) are the most commonly used options and show a good response.^4^ A small number of case reports indicate possible efficacy of intralesional infliximab (off label)^46^, ^47^ or topical ruxolitinib, a JAK1 and JAK2 inhibitor (off label).^48^

**TABLE 3:** Recommendations for local therapy of necrobiosis lipoidica.

**Recommendation**	**Strength**
Use of topical glucocorticoids, preferably class III‐IV according to Niedner, in the marginal area of inflammatory Necrobiosis lipoidica lesions *is recommended* as primary topical treatment.	↑↑
Off‐label use of tacrolimus ointment *is recommended* as an alternative to topical glucocorticoids for anti‐inflammatory topical treatment.	↑↑
Topical use of ruxolitinib (off label) *may be considered*.	0
Intralesional use of glucocorticoids *may be considered* in cases of insufficient response to other treatments.	0
Intralesional use of infliximab (off label) *may be considered* if other treatment options have failed.	0

↑↑ – wird empfohlen, ↑ – kann empfohlen werden, 0 – kann erwogen werden

↑↑ – is recommended, ↑ – may be recommended, 0 – may be considered

### Glucocorticoids

Topical glucocorticoids have been the therapeutic standard for decades.^2^, ^4^ Even though there are no prospective, randomized, controlled clinical trials proving the efficacy of glucocorticoids in NL, experts agree that initial treatment should be attempted with topical glucocorticoids.^4^ Due to the granulomatous character and the depth of the inflammatory infiltration, high‐potency glucocorticoids should be preferred and, where appropriate, applied in conjunction with occlusion.^4^ If the therapeutic effect of topical glucocorticoids remains unsatisfactory even with occlusion, intralesional application of glucocorticoids (such as for example triamcinolone acetonide) may be commenced.^49^

### Tacrolimus (off label)

Tacrolimus is a calcineurin inhibitor which has only been used successfully in case report series.^50^, ^51^, ^52^ Due to its favorable side effect profile, topical tacrolimus is frequently used as a second‐line or third‐line treatment in clinical practice, with occlusion if required.^4^, ^29^

## LIGHT AND LASER TREATMENTS

### UV therapies

Ultraviolet (UV) radiation therapies for NL account for the highest documented number of patients treated. A major advantage of UV treatment is the reduced potential for skin atrophy as compared with glucocorticoids. Disadvantages include time expenditure for the patient and the fact that UV treatment is no longer widely available in dermatological practices. The possible, albeit rare, occurrence of squamous cell carcinomas in NL lesions must also be considered.

The largest body of data in NL treatment exists for PUVA treatment with topical or systemic application of a photosensitizer (usually 8‐methoxypsoralen) and subsequent UV‐A irradiation. This treatment is therefore recommended in the S1 guideline. In NL patients, it is mostly carried out as cream or bath PUVA. A study with 30 patients treated with cream PUVA showed healing or improvement in more than half of the patients. Patients who showed no response or a worsening of symptoms had on average received a lower UV dose.^6^ Other authors also reported improvement or healing in a majority of treated NL patients.^53^, ^54^ Application of UVA1 alone also showed a benefit in 50–66% of treated patients according to published studies,^55^, ^56^ so this form of treatment may also be considered.

### Other physical therapies

When considering photodynamic therapy (PDT), we must keep in mind that low penetration of the radiation source and the photosensitizer (usually δ‐aminolevulinic acid) limit the effect, which constitutes a disadvantage in view of the deep‐reaching inflammatory processes in NL. This might explain why eleven out of 18 patients treated with PDT did not show any improvement of NL symptoms.^5^ In 65 NL patients, prior curettage led to improved penetration of the compound, so complete healing was achieved in 66% and a reduction of the affected area by 80–99% was achieved in an additional 19% of patients.^57^ Daylight PDT has also been used successfully in NL.^58^ PDT may thus be considered.

A review published in 2020 offered a systematic overview of the available evidence on light and laser therapies from various publications.^59^ PUVA was considered the most effective treatment, followed by PDT. Procedures such as pulsed laser treatment, UVA1 treatment, or CO_2_ laser were considered less effective.

### Compression therapy

Compression therapy of the lower legs is traditionally a pillar of conservative treatment in NL patients.^60^ The approaches discussed in this context include reduction of edema, improved microcirculation, and anti‐inflammatory effects. Chronic venous insufficiency (CVI), which has been reported as a potentially increased comorbidity in NL patients, may also constitute a possible trigger.^61^ Compression therapy is an important treatment option with few side effects which should be performed in all patients with NL on the lower legs after possible contraindications have been excluded.

### Surgical treatment

The successful complete excision of skin changes in NL and subsequent defect coverage using skin transplants has been described in several publications.^61^, ^62^, ^63^, ^64^ These surgical procedures were in some cases performed using porcine material and/or artificially grown skin transplants. Due to the invasive nature of the procedure, it remains limited to individual lesions in necrobiosis lipoidica.

### Wound treatment in ulcerated necrobiosis lipoidica

There is no specific wound treatment indicated for NL patients with ulceration.^65^ In these cases, it is recommended to use the phases of wound healing as a guide and to consider factors such as pain, amount of exudate, and local signs of inflammation individually. The M.O.I.S.T. concept offers a helpful guideline in this regard.

### Systemic treatments

Systemic treatment is indicated mainly for NL patients with severe disease and/or ulceration. The therapeutic approach is predominantly aimed at fighting inflammation. Investigating inflammatory activity in the skin lesions is therefore the first step. If no inflammatory activity is detected and the remaining lesions are atrophic but stable in size, the patient must be informed that no resolution of the existing skin lesions can be expected. The majority of reported effects of systemic treatment are based on case reports. Furthermore, there are no clear assessment criteria on what constitutes “improvement” of NL lesions under systemic treatment for evaluation of the published data. For ulcerated lesions, healing of the ulcers has been reported as a measure of success.

### Systemic glucocorticoids

Use of systemic glucocorticoids in the inflammatory stage of NL is widely recognized in clinical practice.^4^, ^29^ However, the influence of steroids on glucose metabolism in diabetic patients must be taken into account.

### Fumaric acid esters (off label)

Fumaric acid esters offer a multitude of immunomodulating properties affecting many cell types in the blood and tissue, such as leukocytes and keratinocytes but also endothelial cells.^67^ Data from a prospective, non‐randomized, non‐controlled study with fumaric acid esters are available.^68^ Eighteen patients received systemic treatment with fumaric acid esters for a minimum of six months. Therapeutic success was assessed clinically, histologically, and with 20‐Mhz ultrasound. A significant improvement in clinical scores was observed under this treatment. In clinical practice, fumaric acid esters are currently used in a number of NL cases, with good response rates.^4^ They are thus recommended by expert consensus and considered effective.^29^

### Ciclosporin (off label)

Based on broad experience with the calcineurin inhibitor ciclosporin in dermatological diseases, use of ciclosporin may be recommended for severe cases of NL after failure of first‐line treatments.^69^, ^70^

### Antimalarial medications (off label)

Several case reports on the successful use of chloroquine and hydroxychloroquine have been published.^70^, ^71^, ^72^ A case series with a total of eight patients showed significant improvement in seven cases.^73^ Due to low cost of the therapy and extensive experience in its use and monitoring, systemic treatment with (hydroxy)chloroquine may be considered.

### Biologicals (off label)

Successful treatment with the TNF‐α inhibitors infliximab, etanercept, and adalimumab in NL patients has been reported in several instances.^74^, ^75^, ^76^, ^77^ Most of these cases were patients with ulcerated and treatment‐refractory NL. Due to the broad experience with these compounds, TNF‐α inhibitors can be recommended for severe, treatment‐refractory cases of NL. Biologicals with other target structures such as ustekinumab (anti‐IL12/IL‐23) have also been used successfully.^78^, ^79^ A case series with secukinumab (anti‐IL‐17) in three patients showed good response.^80^

### Janus kinase inhibitors (off label)

There are a number of Janus kinase (JAK) inhibitors that have been successfully used in individual cases of NL.^81^, ^82^, ^83^, ^84^ In one patient treated with a JAK2 inhibitor for polycythemia vera, improvement of NL was noticed as an additional finding. A 25‐year‐old female patient with type‐1 diabetes and a long‐term history of NL with ulceration and lack of therapeutic response to various treatments was successfully treated with tofacitinib. Combination therapy with intralesional glucocorticoids resulted in further improvement.

### Other systemic treatment approaches (off label)

Although dapsone has been used successfully in other granulomatous diseases, there are little promising data on its use for NL. In an analysis of 98 NL patients, a quarter were treated with dapsone, with a good response reported in only three cases.^4^ In an expert interview in Germany, 43% of dermatologists recommended dapsone for NL treatment.^29^ Thus, dapsone can at least be listed as a medication of 2^nd^ or 3^rd^ choice.

Pentoxifylline has anti‐inflammatory properties and can improve microcirculation. There are some individual case reports on the use of pentoxifylline in NL,^85^, ^86^ both in patients with ulcerated disease and those with lesions in unusual locations. Based on these data, use of pentoxifylline may be considered.

A multitude of substances such as mycophenolate mofetil, doxycycline, thalidomide, clofazimin, colchicin, nicotinamide, or tranilast have been used successfully for treating NL in individual cases or small case series. Based on the lack of consistent data, a recommendation cannot be given for these compounds.

**FIGURE 1:**
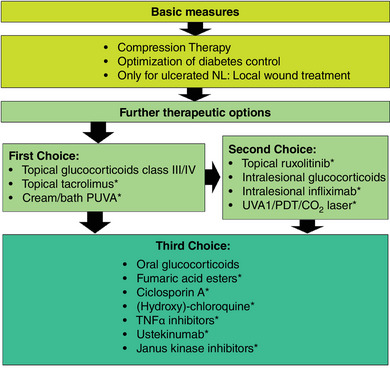
Flow chart of the most important therapy recommendations for NL. *off label

Figure 1 summarizes all recommended therapeutic options.

### Special considerations in children and adolescents

Reports on NL in children and adolescents are mainly available as individual case reports.^87^ Remarkably, almost all of the affected children have type‐1 diabetes with insufficient metabolic control. Topical therapy including high‐potency glucocorticosteroids or calcineurin inhibitors are the preferred treatment for minors. Otherwise, the same recommendations for the diagnosis of associated diseases apply to children as to adults.

### Limitations of this guideline

Randomized, controlled clinical trials (RCT) are not available for any NL treatment; and the number of prospective observational studies is also small. In addition, there is a lack of unambiguous criteria on how to assess improvement of NL lesions with treatment. In view of the current literature, we must assume a bias in favor of positive therapeutic responses, since successful individual case reports have a higher chance of being published.^88^

### Clinical practice summary

Altogether, the scientific database on NL is insufficient and there is a need for further research. It is also remarkable that the available studies have only rarely differentiated between ulcerated and non‐ulcerated forms of the disease as a criterion. The pathophysiology of NL remains unknown. Findings from effective therapeutic measures may form the basis for further research in this area. Since NL patients frequently report a high level of suffering, it is important to be familiar with the limited scientific knowledge available and to take this into account in corresponding treatment approaches.

## CONFLICT OF INTEREST STATEMENT

Listed in the long version of this AWMF guideline (AWMF Registry No.: 013‐096, 2024).
